# Supplementary material for: TCR Clonality and Genomic Instability Signatures as Prognostic Biomarkers in High Grade Serous Ovarian Cancer
Source: Cancers (Basel). 2021 Aug 31;13(17):4394. doi: 10.3390/cancers13174394 (PMC8430641; doi:10.3390/cancers13174394)

Supplementary Table S1: Details of BRCA mutations in the TCGA and CGFL cohorts.

BRCA mutations	Type of mutation	
	TCGA cohort	CGFL cohort
BRCA 1	c.4484+1G>C; c.2438del; c.3795_3796insTT; c.3465_3466insATCTAACA; c.331G>T ; c.346G>T ; c.1557del ; c.3649_3650insTAAGTTCT ; c.3562_3563del; c.2398A>T ; c.5278-1G>T ; c.5129del ; c.1292T>G ; c.882del ; c.1A>G; c.1421T>G	c.5159G>A ; c.1961del; c.181T>G
BRCA 2	c.9831del; c.8716G>T; c.8090_8105del; c.9513_9516del; c.2133C>A; c.5645C>A; c.3688del; c.4218_4221del	c.2539A>T

Supplementary Table S2: Description and association of CNV signature with oncogenes or tumor suppressor genes and immune cell populations.

CNV signatures	*Description*	*Oncogenes or tumor suppressor genes*		*Immune cell popultations*
		Amplification	*Deletion*	
CNV signature 1	Associated with poor prognosis. Related to oncogenic RAS-MAK pathway	AR, CCND3, CCNE1, CD274, CDK6, EGFR, EPHA3, ESR1, JAK2, JUN, MET, MITF, MYC, MYCN, NKX2-1, PAK1, REL, SOX2	-	Associated with an increase of abundance of myeloid dendritic cells.
CNV signature 2	Associated with poor prognosis. Related to tandem duplication through CDK12 inactivation	CD274, CDK4, CDK6, EGFR, ERBB2, FGFR2, JUN, MDM2, MDM4, MITF, MYC, MYCN, REL, SYK, TERT	-	Associated with a decrease of abundance of myeloid dendritic cells.

CNV signature 3	Associated with good prognosis Associated to BRCA 1-2 related HRD	AKT2, AR, AURKA, BCL2, CCND1, CCND2, CCND3, CCNE1, CD274, CDK4, CDK6, EGFR, EPHA3, ESR1, FGF3, FGF4, FGFR1, FGFR2, JAK2, JUN, MDM2, MDM4, MET, MITF, MYC, MYCN, NKX2-1, PAK, REL, RICTOR, SOX2, TERT	PTEN	-
CNV signature 4	Related whole genome duplication due to failure of cell cycle.	AKT2, AR, AURKA, BCL2, CCND1, CCND2, CCND3, CCNE1, CD274, CDK4, CDK6, EGFR, EPHA3, ERBB2, ESR1, FGF3, FGF4, FGFR1, FGFR2, JAK2, JUN, MDM2, MDM4, MET, MITF, MYC, MYCN, NKX2-1, PAK1, REL, RICTOR, SOX2, SYK, TERT	-	-
CNV signature 5	Correlated with number of chromothriptic like events.	AR, AURKA, CDK6, EGFR, JUN, NKX2-1, RICTOR, SOX2, TERT	-	-
CNV signature 6	Associated with focal amplification due to failure of celle cycle control	AKT2, AR , AURKA, BCL2 , CCND1, CCND2, CCND3, CCNE1 , CD274, CDK4, CDK6, EGFR, EPHA3, ESR1, FGF3, FGF4 , FGFR1, JAK2, JUN, MDM2, MDM4, MET, MITF, MYC, MYCN, NKX2-1, PAK1, REL, RICTOR, SOX2, TERT	-	Associated with an increase of abundance of B lineage cells and a decrease of abundance of NK cells and myeloid dendritic cells.
CNV signature 7	Associated with good overall survival. Associated with non BRCA 1-2 related HRD.	AKT2, AR, AURKA, CCND2, CCND3, CCNE1, CDK4, CDK6 , EGFR, EPHA3, ERBB2, ESR1, FGFR2, JUN, MDM2, MDM4, MET, MYC, RICTOR, SOX2, TERT	-	-

The relation between CNVs of 43 available oncogenes or tumor suppressor genes (amplification vs no amplification and deletion vs no deletion) and each of the CNV signatures expressions (Low vs High) using the Chi-2 test. All adjusted p-value less than 0.1 were considered statistically significant

Supplementary Table S3: Univariate Cox model for overall survival and CNV signatures in the CGFL cohort.

Variables	*HR*	*95%CI*	*P-value*	*Adjusted* P-value*
CNV signature 1	1.07	[0.15 ; 7.39]	0.95	0.95
CNV signature 3	0.4	[0 ; 48.88]	0.71	0.85
CNV signature 4	7.48	[0.06 ; 949.93]	0.42	0.85
CNV signature 5	0.001	[0 ; 171762638959.73]	0.68	0.85
CNV signature 6	71.9	[0 ; 419179946.36]	0.59	0.85
CNV signature 7	0.56	[0.03 ; 11.62]	0.71	0.85

*p-values were adjusted using Benjamini-Hochberg FDR correction.
HR, hazard ratio; CI, confidence interval.
CNV signature 2 was not expressed in the CGFL cohort.

Supplementary Figure S1: Flowchart for the TCGA cohort.

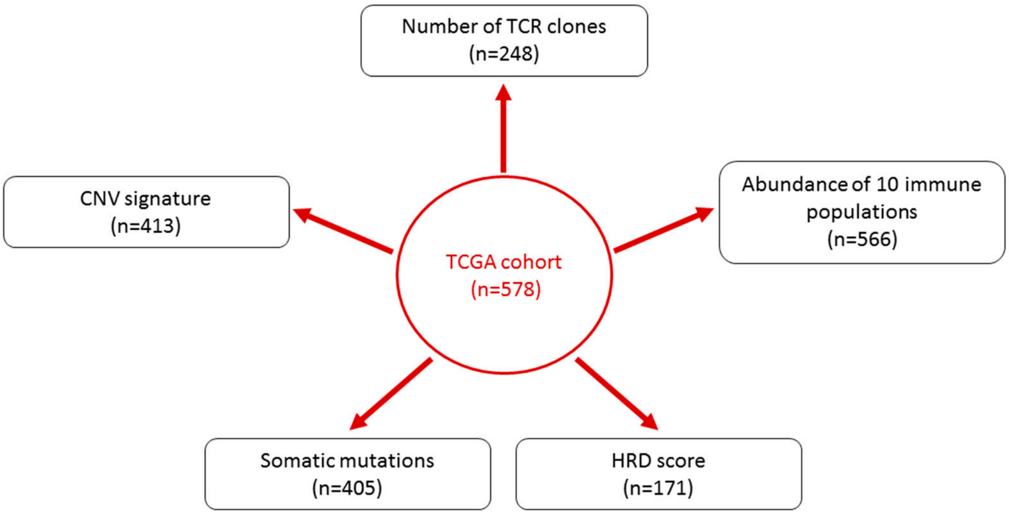

Supplementary Figure S2: Association between HRD profile, immune and genomic landscape and overall survival. (A) Kaplan-Meier curves with patients stratified according to the HRD profile and the abundance of neutrophils for overall survival in the TCGA cohort. (B) Heatmap showing the correlation matrix between HRD score, CNV signatures, immune populations and TCR clonality in the TCGA cohort; correlations were calculated with Pearson's correlation coefficient and p-values <0.05 are represented by a star. (C) Kaplan-Meier curves with patients stratified according to the HRD profile and CNV signature 7 proportion in the CGFL cohort. There are no patients with HRD/CNV7Low tumors. TCR: T Cell receptor, CNV: Copy Number Variant.

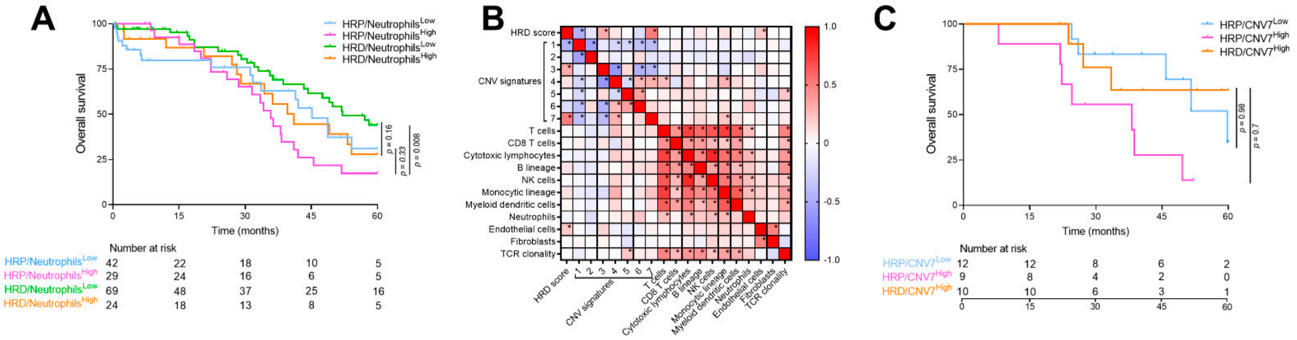

Supplement: Supplementary file 1 [file cancers-13-04394-s001.zip › cancers-1336774-supplementary.pdf]
